# Organizational leadership competencies for effective and equitable public health governance in Canada

**DOI:** 10.3389/phrs.2026.1609387

**Published:** 2026-08-04

**Authors:** Garima Talwar Kapoor, Shinjini Mondal, Kian Rego, Madelyn Law, Erica Di Ruggiero

**Affiliations:** 1 University of Toronto, Toronto, ON, Canada; 2 McMaster University, Hamilton, ON, Canada; 3 Brock University, St. Catharines, ON, Canada

**Keywords:** competencies, governance, leadership, organizational, public health

## Abstract

**Background:**

Organizational-level leadership competencies are critical for the development and governance of resilient public health systems that can endure and thrive in an ever-evolving economic, social, and political context.

**Analysis:**

A mixed-methods research study identified 20 organizational leadership competencies for public health in Canada, across eight domains, including: systems thinking (e.g., leaders are able to adapt, are nimble, and innovate), policy development/implementation/evaluation (e.g., leaders engage multiple actors in policy) and resource stewardship (e.g., leaders develop a resource allocation strategy that balances efficiency and equity).

**Policy Options:**

Recommendation: Governments across Canada should undertake legislative and regulatory efforts to implement organizational-level leadership competencies in public health organizations to strengthen system-level governance. Status quo: Without a systemic approach, some organizations will continue to undertake actions that strengthen their governance, while others will not. This could exacerbate inequities in population health outcomes and organizational performance.

**Conclusion:**

Strengthening organizational leadership competencies is critical for the effective and equitable governance of public health organizations in Canada. Failure to do so could undermine their resilience in a resource-constrained and changing context.

## Background

The COVID-19 pandemic brought into sharp relief the need to strengthen public health systems across Canada and the world. To be effective and resilient, public health systems require strong leadership for adequate and stable financing, high-quality and accessible evidence, interoperable information systems, effective governance, and a competent workforce [1].

While much focus is placed on individual leadership competencies in public health [2], from academic research to workplace learning and workforce development, less is understood about the organizational-level leadership competencies required for public health governance. Therefore, research is needed to define these competencies to inform public health policy and practice. For the purposes of this brief, public health and its institutions are defined as follows: “Public health is an organized effort by society, primarily through its public institutions, to improve, promote, protect and restore the health of the population through collective action.” (Pan-American Health Organization, as cited in Azari and Borisch, 2023).

Organizational-level leadership focuses on the collective leadership qualities between members of a team to advance the mandate, goals, and values of an organization. These competencies are critical for the development of resilient public health systems that can endure and thrive in an ever-evolving economic, social, and political context. A resilient, equitable, and sustainable public health system requires effective governance across levels of government (e.g., federal, provincial/territorial), within public health organizations, and among multiple sectors outside of health [3].

This policy brief provides an overview of the organizational-level leadership competencies for effective and equitable public health governance in Canada. It summarizes a multi-phased, mixed-methods research study and situates/analyzes the findings from this research in the literature. The brief also identifies possible policy options that could advance the development and implementation of these competencies in public health organizations.

## Analysis

### Methods from novel research in Canada

Findings from a mixed-methods research study identified 20 organizational-level leadership competencies for effective and equitable public health governance in Canada, across eight domains [4]. The first two phases of the study consisted of a scoping review and key informant interviews, and detailed results are reported elsewhere [5, 6]. Building on the findings from the scoping review and interviews, a two-round modified Delphi survey was conducted with public health experts across Canada to develop consensus on the domains and competencies of organizational-level leadership for public health governance. A non-random sample of expert panel members was identified by the research team through actor mapping. There were 62 survey participants in Round 1. Round 2 had a 72.58% retention rate (n = 45). Delphi participants represented Canada’s regional diversity and varied in leadership position and experience. For example, half of the participants represented Alberta, Saskatchewan, Manitoba and Ontario, approximately half represented local public health organizations, a diversity of expertise (e.g., Indigenous health, chronic disease); and leadership positions (e.g., Chief Medical Officer of Health, General Manager/program manager). Some socio-demographic data were collected but not reported to maintain the anonymity of the respondents. The third round of the Delphi technique involved a deliberative dialogue. Over the course of a half-day session, 13 public health experts, representing diverse disciplinary and organizational perspectives, were convened to finalize the list of competencies and generate policy options for the uptake of competencies by public health organizations in Canada.

**FIGURE 1:**
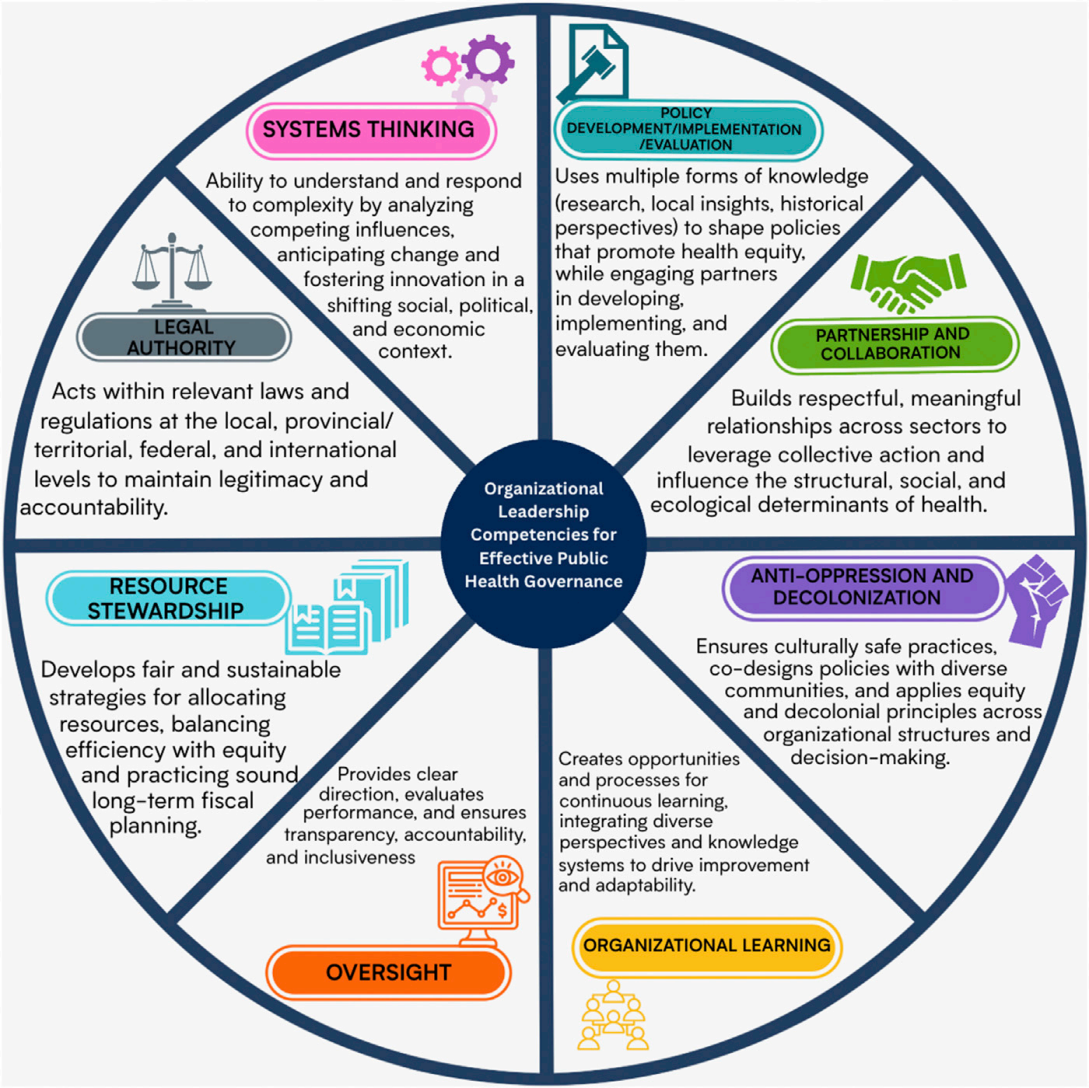
Organizational leadership competency wheel for public health governance in Canada. Source: created by the authors (modified Delphi study, Canada, 2025–2026).

The multi-pronged research approach led to the identification of the following 20 organizational-level leadership competencies, across eight domains, for effective and equitable public health governance in Canada [4] (Figure 1).

### Findings in relation to broader literature

The organizational leadership competencies identified in this study contribute to the global body of comparative evidence on effective public health system governance and are relevant to the Canadian context. For example, research emphasizes stewardship, strategic direction, accountability, trust, and coordination across actors as core health system governance capacities [7–9]. These governance functions are reflected in the competencies identified here, particularly those related to strategic alignment, relationship management, ethical decision-making, and cross-sector collaboration. Importantly, this study is one of the few to identify the need for organizational governance and to advance the domain.

Furthermore, effective governance depends on adaptive, collective leadership capable of navigating complexity and uncertainty. The findings underscore leadership as a shared organizational function requiring enabling structures, cultures, and processes. This requires public health leaders to move beyond organizational silos, strengthen partnerships, and foster learning and innovation [10].

**FIGURE 2:**
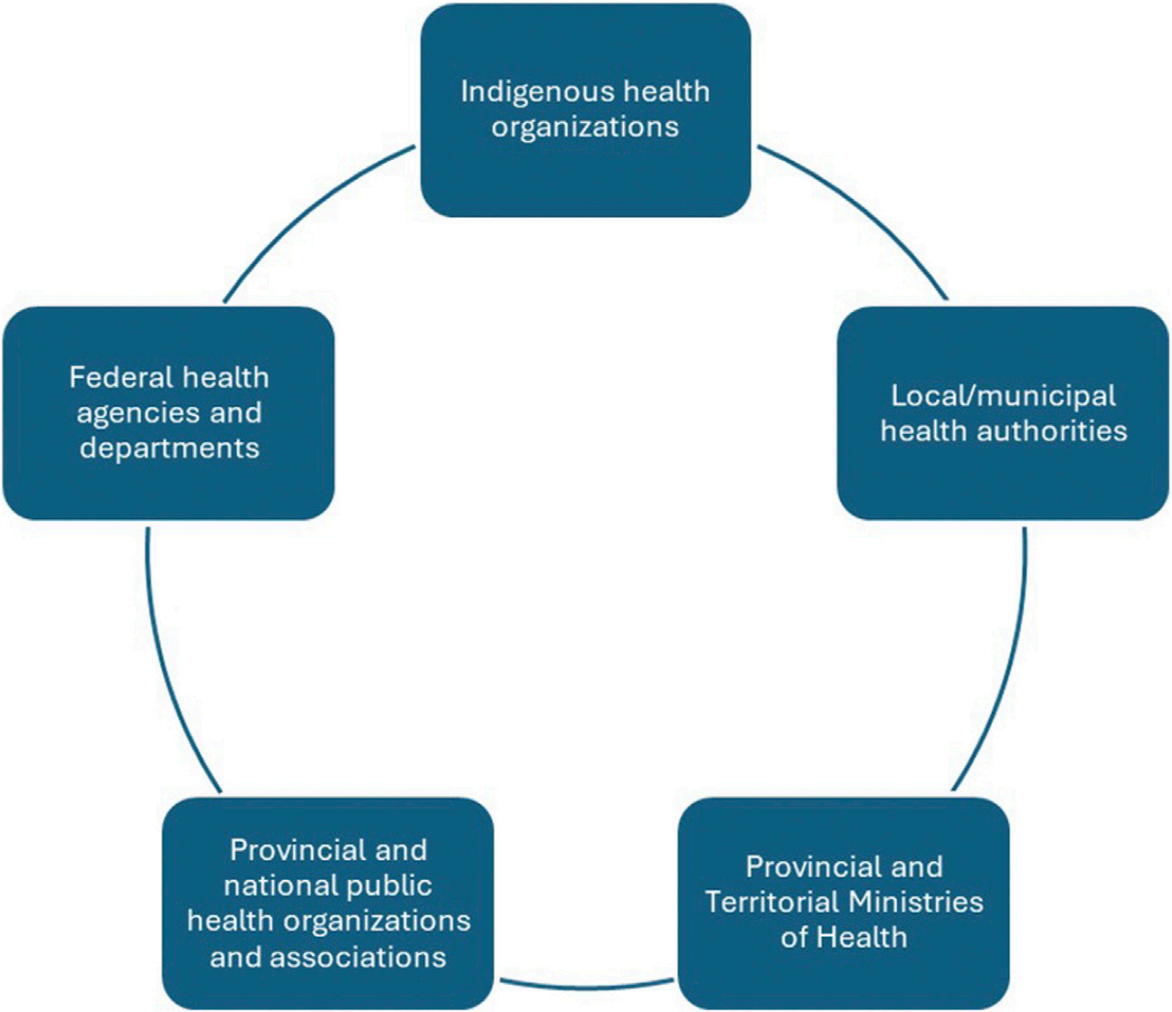
Canada’s multi-level and multi-actor system. Adapted from the Chief Public Health Officer of Canada’s Report on the State of Public Health in Canada (2021).

Given Canada’s federated system of public health, this mixed-methods, multi-phase study reflects the importance of focused evidence on the organizational-level leadership competencies needed for effective and equitable public health governance in Canada. Figure 2, adapted from the Chief Public Health Officer of Canada’s Report on the State of Public Health in Canada (2021), illustrates Canada’s public health governance operating in a federated, multi-level, and multi-actor context. It reflects the distribution of roles across governments, Indigenous partners, and non-governmental actors, and the horizontal and vertical relationships required for effective public health action.

## Policy options

### Context

The results of the study at the heart of this brief point to the importance of understanding and examining leadership competencies collectively—that is, it reflects the adage that the whole is greater than the sum of its parts. While significant attention is paid to individual leadership development and growth in public health training, understanding the leadership competencies needed across all levels of an organization in a collective and complementary way can help advance the mandates of public health organizations more effectively and sustainably.

There is a risk that public health organizations do not meet their mandates and expectations of improving population health and equity outcomes if a concerted effort on developing the requisite organizational-level competencies is not undertaken. This is especially true given the quick changes in the economic, social, and political context in which public health organizations in Canada operate.

The policy options discussed below reflect the mixed-methods research at the center of this brief, including the deliberative dialogues undertaken with public health leaders across Canada.

### Recommended policy option

The study insights at the core of this brief suggest that governments across Canada should undertake legislative, regulatory, and/or policy efforts to develop organizational-level leadership competencies in public health organizations and strengthen system-level governance.

As Canada is a federated country, the federal, provincial/territorial, and municipal/local governments are responsible for public health. While public health responsibilities vary by and within jurisdiction, all levels of government need to be engaged to strengthen organizational-level leadership competencies in public health.

Given the various governments and jurisdictions that play a role in public health in Canada, it is important to advance policy changes that strengthen coordination and shared stewardship of public health in Canada. The development of organizational-level leadership competencies provides an opportunity to articulate a cohesive and unified view of what is required across all levels of public health organizations to achieve effective public health governance. This could be one step in advancing the development of a public health “system” in Canada.

Legislative and regulatory mechanisms currently exist to outline the expected standards and mandates of public health organizations in jurisdictions across Canada. These mechanisms could be further strengthened by articulating the organizational-level leadership competencies for effective and sustainable public health governance. Subsequently, government-led performance monitoring to understand how public health organizations are progressively developing, strengthening, and implementing these organizational-level competencies could help incentivize leadership to focus on this issue. At the same time, implementation of these competencies would enable governments across Canada to make regulatory changes that reflect their jurisdictional contexts.

The COVID-19 pandemic exposed the strengths and gaps in Canada’s public health infrastructure and governance. There will likely be public support to undertake activities that strengthen public health governance in Canada. Given the economic headwinds facing Canada today (e.g., trade conflict with the United States), economic uncertainty could further affect population health and equity outcomes. As such, all efforts that could help stabilize, let alone improve, population health outcomes in this context are necessary.

A focused effort to develop and implement these competencies may be haphazard—that is, not all public health organizations will, or have the resources to, undertake their development and implementation concurrently. Public health organizations may seek funding to further develop these organizational-level competencies, but given the resource constraints faced by all governments across Canada, it is unlikely that organizations will be given additional fiscal resources to develop these competencies. This could exacerbate inequities in organizational performance and at the population level.

Importantly, however, the recommended option asks jurisdictions in Canada to advance changes that would create a regulatory framework for organizational-level leadership competencies in public health governance. Once these legislative, regulatory, or policy changes are made, they could create the North Star for the competencies required for effective and equitable public health governance in Canada, and allow for their implementation (e.g., whether phased or targeted on particular competencies) depending on jurisdictional contexts.

Furthermore, while the organizational-level competencies identified through the research are comprehensive, diversity in jurisdictional responsibilities and authorities means that there may be nuances in how different domains and competencies are approached (e.g., competencies for resource stewardship might differ if funding for an organization is dependent on another level of government). Given how novel the concept of organizational-level leadership competencies in public health is, it may take time for jurisdictions across Canada to advance the regulatory framework needed for the development and implementation of the competencies.

### Alternative policy option

Federal public health organizations could communicate the findings of the research to public health training and education institutions (e.g., universities) to encourage the inclusion of these competencies in public health education curricula. This approach could be integrated with existing curricula in public health institutions about individual leadership competencies needed for public health practice. This could help demonstrate both the importance of organizational-level leadership competencies and how they may complement individual leadership competencies.

However, this option could create some costs for universities and other educational institutions, as they may need to revise existing course offerings or create new ones. While this is a low-cost option for the government (financially and in terms of time on their policy agenda), organizational culture matters and affects how these organizational-level competencies are developed and implemented. It is difficult to know whether education about these competencies outside of an organizational context can help build organizational-level leadership competencies for effective public health governance.

### Status quo

A continued focus on individual-level leadership competencies in public health educational institutions or workplaces alone will not improve the governance of public health organizations. Failure to develop and implement a systemic approach to organizational-level competencies means that some public health organizations will continue to undertake actions that strengthen their governance, while others will not. This could exacerbate inequities in population health outcomes and organizational performance across jurisdictions in Canada. Furthermore, given the resource constraints that governments across Canada are facing, along with the heightened public scrutiny of public health since the COVID-19 pandemic, there is a risk that public health will be marginalized as a sector in Canada. Given ongoing public health governance changes across Canada, this is an opportune time to consider the inclusion and implementation of organizational-level competencies to ensure effective public health governance.

Table 1 provides a comparative summary of the policy options outlined in this brief.

**TABLE 1:** Comparative table of policy recommendations (Canada, 2025–2026) [11].

Option	Pros	Cons	Barriers to implementation	Expected outcomes
Legislative/regulatory/policy changes	• Articulate a cohesive and unified view of what is required to advance organizational-level leadership competencies for public health governance across Canada • Mechanisms exist across jurisdictions to make these changes	• Only sets out the regulatory framework for organizational-level leadership competencies in public health—does not necessitate their adoption	• Implementation of organizational-level competencies depends on jurisdictional and organizational resources (e.g., fiscal, labor) • The economic context is evolving, and fiscal constraints facing public health organizations are deepening; implementation of the competencies may vary across jurisdictions	• As competencies are integrated into legislative/regulatory or policy frameworks, they will start to be adopted over time • Will strengthen equitable and effective public health governance across jurisdictions in Canada
Integration into educational curricula	• Enables academic and training institutions to teach organizational-level competencies in their curricula, ensuring that current (e.g., staff) and future (e.g., students) members of the public health workforce are prepared to apply said competencies for effective public health governance, both as individuals and within organizations	• Does not advance systemic change and enables academic and training institutions to adopt the teaching of these competencies in curricula on an *ad hoc* basis	• As a novel area of work, many academic and training institutions will first need to be made aware of the potential of organizational-level leadership competencies and what they aim to achieve	• Canada’s public health workforce is increasingly becoming aware of organizational-level leadership competencies in public health governance and how they may be advanced to support equitable and effective governance
Status quo	• Does not require public health organizations and governments across Canada to expend any resources (e.g., labor) on advancing organizational-level leadership competencies, enabling them to allocate resources to competing public health priorities	• Impairs the ability to systematically strengthen public health governance in Canada	• N/A	• Continues to see public health organizations across Canada struggle to advance their mandates in an increasingly complex social and economic environment • Impairs public health outcomes in the long run

## Conclusion

Strengthening organizational-level leadership competencies is timely and critical for the effective and equitable governance of public health organizations in Canada. The multi-pronged research study described above led to the identification of 20 organizational-level leadership competencies across eight domains, including systems thinking, resource stewardship, and legal authority.

Legislative and regulatory mechanisms could be employed across Canada to articulate not only the mandates and standards expected of public health organizations, but also the organizational-level competencies required to help improve governance. Jurisdictions across Canada must undertake concerted efforts to advance changes in their regulatory framework to encourage public health organizations and their workforces to adopt and strengthen their organizational-level leadership competencies. In doing so, public health organizations will be better equipped to respond to the challenges of the evolving economic and social context in Canada. On the other hand, failure to develop and implement the requisite organizational-level competencies noted could impair the ability of public health organizations to remain resilient in a resource-constrained and changing context.

Further research is required to test the implementation of the organizational-level competencies identified in research thus far to understand their applicability and potential to strengthen public health governance in diverse settings across Canada.
